# Social Media Opinions on Working From Home in the United States During the COVID-19 Pandemic: Observational Study

**DOI:** 10.2196/29195

**Published:** 2021-07-30

**Authors:** Ziyu Xiong, Pin Li, Hanjia Lyu, Jiebo Luo

**Affiliations:** 1 University of Rochester Rochester, NY United States

**Keywords:** characterization, COVID-19, social media, topic modeling, Twitter, work from home

## Abstract

**Background:**

Since March 2020, companies nationwide have started work from home (WFH) owing to the rapid increase of confirmed COVID-19 cases in an attempt to help prevent the disease from spreading and to rescue the economy from the pandemic. Many organizations have conducted surveys to understand people’s opinions toward WFH. However, the findings are limited owing to a small sample size and the dynamic topics over time.

**Objective:**

This study aims to understand public opinions regarding WFH in the United States during the COVID-19 pandemic.

**Methods:**

We conducted a large-scale social media study using Twitter data to portray different groups of individuals who have positive or negative opinions on WFH. We performed an ordinary least squares regression analysis to investigate the relationship between the sentiment about WFH and user characteristics including gender, age, ethnicity, median household income, and population density. To better understand the public opinion, we used latent Dirichlet allocation to extract topics and investigate how tweet contents are related to people’s attitude.

**Results:**

On performing ordinary least squares regression analysis using a large-scale data set of publicly available Twitter posts (n=28,579) regarding WFH during April 10-22, 2020, we found that the sentiment on WFH varies across user characteristics. In particular, women tend to be more positive about WFH (*P*<.001). People in their 40s are more positive toward WFH than those in other age groups (*P*<.001). People from high-income areas are more likely to have positive opinions about WFH (*P*<.001). These nuanced differences are supported by a more fine-grained topic analysis. At a higher level, we found that the most negative sentiment about WFH roughly corresponds to the discussion on government policy. However, people express a more positive sentiment when discussing topics on “remote work or study” and “encouragement.” Furthermore, topic distributions vary across different user groups. Women pay more attention to family activities than men (*P*<.05). Older people talk more about work and express a more positive sentiment regarding WFH.

**Conclusions:**

This paper presents a large-scale social media–based study to understand the public opinion on WFH in the United States during the COVID-19 pandemic. We hope that this study can contribute to policymaking both at the national and institution or company levels to improve the overall population’s experience with WFH.

## Introduction

### Background

COVID-19 was first reported in China and then spread worldwide and has caused 22.3 million confirmed cases and more than 373,000 deaths in the United States as on January 11, 2021 [1]. To help prevent the virus from spreading and to salvage the economy, companies and schools nationwide have started work and study from home, respectively. According to a Gartner survey of 880 global human resources executives on March 17, 2020, almost 88% organizations have encouraged or required employees to work from home (WFH) [2]. Barrero et al [3] found that WFH might persist even after the pandemic. Concerns may arise regarding productivity [4], willingness [5], and future trends [3,6] regarding work and study from home.

### Prior Studies

WFH has been a controversial issue that merits closer scrutiny. Palumbo [5] reported that WFH might incur side-effects such as a negative impact on work-life balance. This would lead to negative opinions on WFH when people tweet about it. Other studies have focused on specific categories. A survey of employees in Lithuania [7] reported that female employees appreciate WFH more than male employees because female employees can enjoy a healthier lifestyle, while male employees worry about career constraints. However, another survey conducted in the United States [4] shows “a gender gap in perceived work productivity”: before implementing WFH, female and male employees reported the same level of self-rated work productivity. After transitioning to WFH, male employees performed with higher productivity than female employees [4]. Regarding age, people in their 40s have more negative opinions on WFH because of their unfamiliarity with teleworking. People aged 30-39 years have the most positive opinions because they can enjoy time with their families and they are already accustomed with new technologies for teleworking [7]. Previous studies [3,7,8] also show that opinions concerning WFH vary across different socioeconomic groups. A similar social media study of public sentiments on WFH has been conducted in the United Kingdom [9] and reported that more than 70% of tweets concerning WFH expressed a positive sentiment, with the main topics including “traffic,” “drink,” and “e-commerce.”

Similar approaches have been implemented by researchers who mined Twitter posts on public attitudes toward face masks through natural language processing [10] using the Valence Aware Dictionary and Sentiment Reasoner (VADER) model [11] to perform sentiment analysis. Moreover, Twitter data have been used to study many different aspects of COVID-19, such as mining of overall public perception of COVID-19 [12], college students’ attitudes toward the pandemic [13], people’s attitude toward potential COVID-19 vaccines [14,15], sentiment analysis among pregnant women during quarantine [16], and monitoring of depression trends on Twitter during the COVID-19 pandemic [17]. These studies have used VADER for sentiment analysis, and most of them also include a time-series analysis. In addition, we followed the practice of using latent Dirichlet allocation (LDA) [18] to identify topics among a large text corpus. The M3-inference model [10] was used to portray different demographic groups.

### Study Objectives

In this study, we intend to understand public opinions on WFH, using large-scale social media data. Twitter has been a popular social media platform for people, especially in the United States, to express their opinions on what is happening around them. In contrast, the Boston Consulting Group used survey data to study employees’ opinions regarding WFH owing to the COVID-19 pandemic [19]. However, social media data allow an opportunity for conducting a timelier study of many population-level issues on a larger scale [20]. We acquired data with an authorized Twitter developer account using Tweepy. This ensures reliability by acquiring first-hand and sufficient data for the study.

In this study, we also inferred user demographic information using Twitter user information. This is important, since we can carry out an in-depth assessment of the characteristics of those who are more pro-WFH. For example, when we consider gender, we understand that historically mothers have been mostly responsible for caring for children [21]. Therefore, we need information regarding the users’ gender to determine whether there is any difference in sentiment toward WFH between women and men, as WFH would allow female employees to allocate more time to spend with their children.

Our goal is to understand the public opinions on WFH in the United States during the COVID-19 pandemic. In particular, we focused on the following research questions:

1. Who is more likely to tweet about WFH?
2. How does the sentiment of WFH vary across user demographics?
3. Regarding WFH, what do Twitter users mainly discuss? How does the content correlate with the sentiment of WFH?

To summarize, in a large-scale data set of publicly available Twitter posts concerning WFH during April 10-22, 2020, we found that women and older people are more likely to tweet about WFH. On performing ordinary least squares regression analysis, we confirm that sentiment of WFH varies across user characteristics. In particular, women tend to be more positive about WFH than men. People in their 40s are more positive toward WFH than those in other age groups. People from high-income areas are more likely to have positive opinions about WFH.

These nuanced differences are supported by a more fine-grained topic analysis. At a higher level, we found that the most negative sentiment about WFH roughly corresponds to discussions on government policy. However, people express more positive sentiment when discussing topics on “remote work and study” and “encouragement.” Furthermore, topic distributions vary across different user groups.

## Methods

### Methods Overview

In this section, we summarized the data collection process and the methods we used in the analyses. To address research questions 1 and 2, we discuss how we inferred user characteristics and the sentiment in the “Feature Inference” subsection. To address research question 3, we describe how we extracted the topics of tweets in the “Topic Modeling” subsection.

### Data Collection

We collected relevant English-language tweets through the Tweepy stream application programming interface (API) using keywords and hashtag-filtering. The filter keywords and hashtags are “WFH,” “workfromhome,” “work from home,” “#wfh,” and “#workingfromhome.” In total, 553,166 unique tweets with 23 attributes posted by 405,455 unique Twitter users during April 5-26, 2020, were sampled. We attempted to infer the gender, age, and ethnicity of these Twitter users, extract the population density of the area they resided in, and estimate the sentiment of the tweets. There are 405,455 unique users in our data set, 313,815 (77.3%) of whom only tweeted once. After excluding duplicates and users with incomplete features, 28,579 unique Twitter users with all features were included in the data set.

### Feature Inference

#### Sentiment

A normalized, weighted composite score was calculated for each tweet, using VADER [11] to measure the sentiment. The score ranged from –1 (most negative) to +1 (most positive). For validation, we randomly select 194 users’ tweets within 1 month. By manually labeling the sentiment and comparing the sentiment scores with VADER scores, we found that the accuracy was 76%, suggesting that the automatic natural language processing methods we used provide adequate estimates of the sentiment of the tweets. The mean sentiment score was 0.242 (SD 0.448; range –0.967 to 0.984; 25th percentile 0.000, 50th percentile 0.318, 75th percentile 0.617).

#### Age and Gender

We applied the M3-inference model [22] to infer the gender and age of each Twitter user from their profile name, username (screen name), and profile description. Age is binned into four groups: ≤18 years, 19-29 years, 30-39 years, and ≥40 years. The gender distribution of Twitter users is biased toward men (71.8%) [23]. A similar pattern was also observed in our data set, where 57.9% of users are men and 42.1% are women. With respect to age, 37.08% of the users in our data set are older than 40 years, 37.6% are between 30 and 39 years old, 16.5% are between 19 and 29 years old, and the rest are younger than 19 years old. According to a report from the Pew Research Center [24], Twitter users are younger than the average US adult; 21% of adults are aged 18-29 years, 33% are aged 30-49 years, 26% are aged 50-64 years, and 20% are aged ≥65 years. The percentages of adults in the Twitter user population are 29%, 44%, 19%, and 8%, respectively. The pattern in our data set is more similar to that of the distribution of US adults.

#### Ethnicity

To estimate the ethnicity of Twitter users, we applied the Ethnicolr API, which makes inferences on the basis of the last name and first name or just the last name of the Twitter user [25]. In our study, we removed emoji icons, hyphens, unrelated contents, and special characters to extract the last names and applied “census_ln” to infer the ethnicity, which included White, Black or African American, Asian or Pacific Islander, American Indian or Alaskan Native, and Hispanic.

In our data set, White (83.4%) was predominant over other ethnicities, while according to the US Census Bureau [26], White ethnicities accounted for 60.1% of the US population; 7.3% of Twitter users in this study were Asian or Pacific Islanders, while this ethnicity accounts for 6.1% of the US population; 6.5% of Twitter users in this study were Hispanic, while this ethnicity accounted for 18.5% of the US population; 2.5% of Twitter users in this study were Black or African American, while this ethnicity accounted for 13.4% of the US population; American Indian or Alaskan Natives constituted 0.26% of our study’s Twitter user population. According to a report from the Pew Research Center [24], the proportion of individuals by race or ethnicity are almost the same between the US population and Twitter adult users. Interestingly, the proportions of White and Asian or Pacific Islander individuals are much higher than those in the general US population, which could be related to the labor force distributions of these 2 groups. In 2018, 54% of employed Asian and 41% of employed White individuals, compared with 31% of employed Black or African American and 22% of employed Hispanic individuals, worked in management, professional, and related occupations [27], which can most likely be managed from home [28]. Therefore, it is not surprising that there are more White and Asian or Pacific Islander individuals in our data set owing to the disparities in the occupations.

#### Population Density

The USzipcode search engine was applied to extract the population density of each user’s location that Twitter users self-report in their profile information. The population density is categorized as urban (greater than 3000), suburban (1000-3000), and rural (lower than 1000). Finally, 67.4% of users in this study were from urban areas, 14.6% were from suburban areas, and the rest were from rural areas. The majority of the users in our data set were from urban areas, which is consistent with the fact that 83% of the US population resides in urban areas [29]; however, there were proportionally fewer urban users in our data set than in the US population.

#### Income

To investigate the relationship between people’s attitude toward WFH and the gap between high- and low-income areas, we retrieved regional median income from the 2019 American Community Survey. Census API tools were used to extract the median income with an input of city-level user location. The median regional income was US $33,538 (SD US $10,298; range US $3951-121,797; 25th percentile US $28,072, 50th percentile US $31,613, 75th percentile US $36,336).

### Topic Modeling

We used LDA [18] to extract topics from the tweets. In our study, we used the stop words package of the Natural Language Toolkit library, extended with topic-related words (eg, “work” and “home”). To extract the most relevant topics, we only collected nouns, verbs, adjectives, and adverb lemmas. We use the spaCy package to screen all the words of the tweets and only includes those words whose postag is “NOUN,” “ADJ,” “VERB,” or “ADV.” We tuned the hyperparameters with nested looping topic numbers α and β. Finally, we chose *num_topics*=9, α=0.91, β=0.31, and a coherence score C_v_ of 0.379.

## Results

### Sentiment Analysis

As indicated above, Twitter users’ opinions of WFH were slightly positive. We attempted to investigate the relationship between user characteristics and the sentiment of discussions on WFH. We performed ordinary least squares regression analysis on our data set (n=28,579). Descriptive statistics and bivariate correlations are shown in Table 1. Table 2 summarizes the results of ordinary least squares regression analysis.

**Table 1:** Descriptive statistics and bivariate correlation coefficients for study variables^a^.

Variables	Mean (SD)	1	2	3	4	5	6	7	8	9	10
1	0.59 (0.49)										
2	0.09 (0.28)	–.04^b^									
3	0.16 (0.37)	–.21^b^	–.14^b^								
4	0.38 (0.48)	.01	–.24^b^	–.34^b^							
5	0.03 (0.16)	.01^c^	.02^b^	–.00	–.03^b^						
6	0.07 (0.26)	.01	.07^b^	–.02^b^	–.01	–.05^b^					
7	0.01 (0.05)	.02^b^	.03^b^	–.01	–.00	–.01	–.01^c^				
8	0.07 (0.25)	–.00	.02^b^	.04^b^	.03^b^	–.04^b^	.00	–.02^b^			
9	0.25 (0.09)	.04^b^	–.03^b^	–.07^b^	–.01	–.01	.04^b^	.00	–.02^b^		
10	0.67 (0.47)	–.02^b^	.02^b^	.03^b^	.02^b^	–.01	.05^b^	–.00	.03^b^	.05^b^	
11	0.15 (0.35)	.02^b^	–.02^b^	–.02^b^	.01^c^	.01^c^	–.02^b^	.00	.00	.09^b^	–.60

^a^Study variables: 1=gender (0=female, 1=male), 2=age ≤18 years (0=no, 1=yes), 3=age of 19-29 years (0=no, 1=yes), 4=age of 30-39 years (0=no, 1=yes), 5=Black or African American ethnicity (0=no, 1=yes), 6=Asian or Pacific Islander ethnicity (0=no, 1=yes), 7=American Indian or Alaskan Native ethnicity (0=no, 1=yes), 8=Hispanic ethnicity (0=no, 1=yes), 9=income (normalized using MinMaxScaler), 10=urban (0=no, 1=yes), and 11=suburban (0=no, 1=yes).

^b^*P*<.001.

^c^*P*<.05.

**Table 2:** Ordinary least squares regression outputs for public opinions (N=28,579) on working from home against demographics and other variables of interest.

Predictor	Sentiment score	
	*β* (SE)	95% CI
Intercept	0.252^a^ (0.011)	0.231 to 0.273
Gender (0=female, 1=male)	–0.021^a^ (0.006)	–0.032 to 0.010
Age ≤18 years (0=no, 1=yes)	–0.084^a^ (0.010)	–0.103 to –0.064
Age of 19-29 years (0=no, 1=yes)	–0.076^a^ (0.008)	–0.092 to –0.060
Age of 30-39 years (0=no, 1=yes)	–0.022^a^ (0.005)	–0.034 to –0.010
Black or African American ethnicity (0=no, 1=yes)	0.023 (0.017)	–0.011 to 0.066
Asian or Pacific Islander ethnicity (0=no, 1=yes)	0.003 (0.010)	–0.020 to 0.020
Hispanic ethnicity (0=no, 1=yes)	–0.006 (0.011)	–0.027 to 0.016
American Indian or Alaskan Native ethnicity (0=no, 1=yes)	–0.013 (0.052)	–0.115 to 0.088
Income	0.143^a^ (0.031)	0.082 to 0.203
Urban (0=no, 1=yes)	–0.007 (0.007)	–0.021 to 0.007
Suburban (0=no, 1=yes)	–0.004 (0.009)	–0.023 to 0.014
F-statistics	15.25^a^	
R^2^	0.006	
Adjusted R^2^	0.005	

^a^*P*<.001.

#### Women Tended to Be More Positive About WFH

Men were significantly more negative about WFH than women (*P*<.001). This is consistent with the remote work survey report by Fast Company [30]. A more positive sentiment observed among women could be due to the change in working styles [30] and fewer work hours than those of men [31]. A previous survey [7] indicates that women favor WFH from the perspective of a healthier lifestyle.

#### People in Their 40s Are More Positive Toward WFH Than Other Age Groups

Age is another perspective. Results of regression analysis revealed that as age increases, people are significantly more pro-WFH (*P*<.001). This is consistent with the results of the survey conducted by Watkins [32] that generation Z individuals (people aged 8-23 years as of this writing) are more pro-office than millennials (aged 24-39 years). While assumptions exist among older employees, who might be unfamiliar with electronic devices and thus become more pro-office. However, an article in the Financial Times [33] reported contrasting observations. People aged ≥40 years are less likely to be re-employed; hence, they prefer to retain their current jobs while avoiding the risk of being exposed to COVID-19, especially since this group is most vulnerable to COVID-19. Further details regarding these topics will be discussed in the following section. Furthermore, we observed the same pattern as that reported in a survey conducted among employees in Lithuania [7].

#### People in Higher-Income Areas Are More Likely to Have Pro-WFH Opinions Than Those in Lower-Income Areas

Income was significantly correlated with the sentiment toward WFH (*P*<.001). This is concurrent with our finding that people from urban areas would be more pro-WFH, since the regional median income would be higher in big cities [34]. This finding is also in line with that of Barrero et al [3] that high-income workers, in particular, enjoy the perks of WFH.

### Topic Analysis

Further, we attempted to investigate what Twitter users mainly discuss with reference to WFH. In particular, we investigated how the contents of the tweets correlate with the sentiment of WFH. Table 3 shows the 9 topics extracted by the LDA model. We assigned each topic a title on the basis of the top 10 keywords.

**Table 3:** Titles and the top 10 keywords of the topics extracted by the latent Dirichlet allocation model.

Topic#	Topic title	Topic keywords
1	Family activities	dog, try, today, wife, day, last, school, virtual, watch, look
2	Remote work or study	remote, new, time, covid, learn, great, help, many, support, join
3	Quarantine	pandemic, stay, safe, day, go, today, let, see, also, think
4	Dressing	dress, get, enough, adult, day, wear, time, zoom, right, thank
5	Government and policy	money, less, job, option, able, new, remotely, safely, force, take
6	COVID-19 side effects	people, still, do, job, good, say, first, place, probably, go
7	Encouragement	get, lot, world, honestly, fall, reveal, love, week, look
8	Back to office	go, back, know, office, time, feel, quarantine, hour, tip, covid
9	Leasing	office, well, year, instead, couple, permanently, think, lease, renew, similarly

**Figure 1:**
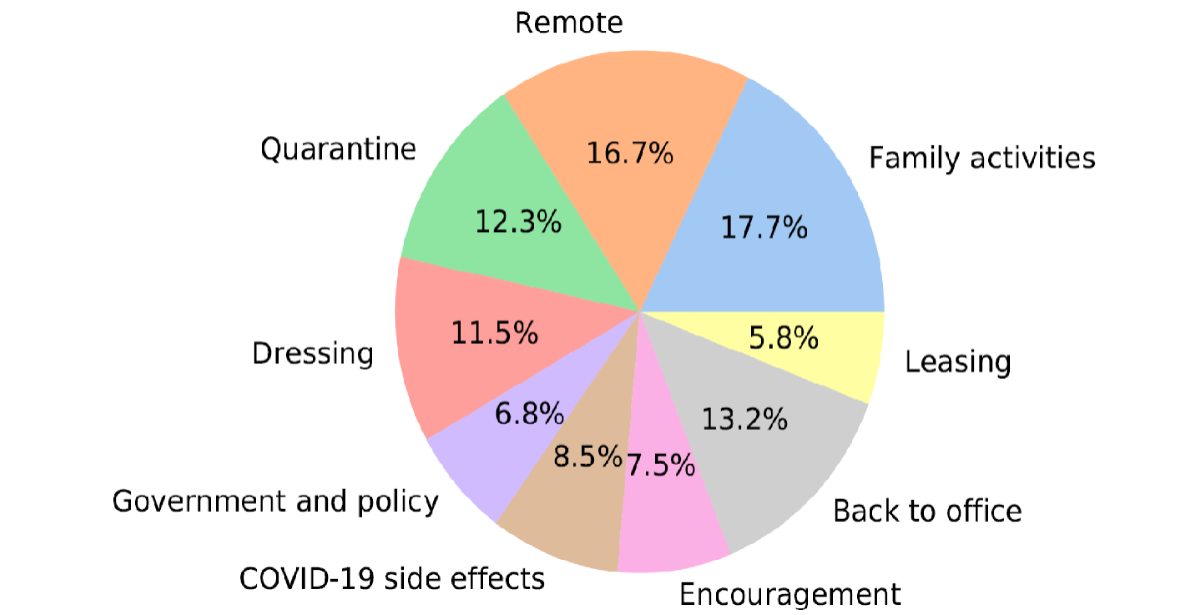
Topic distributions.

Figure 1 shows the proportions of the topics. Topic 1 (family activities) contained the keywords “dog,” “wife,” and “watch” and accounts for 17.7% of the total tweets. Topic 2 (remote work or study) accounts for 16.7% of the total tweets, where people mostly tweeted about remote work and study. Topic 3 (quarantine) contained the keywords “pandemic,” “force,” and “stay.” Topic 4 (dressing) contained the keywords “dress” and “wear,” and most of the tweets based on this topic discuss what people wear when they WFH. Topic 5 (government and policy) accounts for 6.8% of the total tweets and contained the keywords “money,” “job,” and “force”; in this topic, many of the tweets mention the names of governors and express their concerns about WFH-related policies. An example of such a tweet is as follows:

“@GovMurphy #CancelRentNJ If NJ doesn’t cancel rent, the consequences for everyone who can’t work from home will be catastrophic. The bailout money will go straight to landlords instead of feeding people.”

Topic 6 (COVID-19 side effects) contained the keywords “still” and “job” and accounted for 8.5% of the total tweets. In this topic, people mostly complained about the influence of COVID-19, such as “job changing” and “staying home for so long.”An example of such a tweet is as follows：

“I agree. I think the pandemic has shown the disparity of digital access in rural areas, and libraries in these kinds of communities should take note of what these people need so they can provide better services when all of this is over. @Sonaite @BakerChair #SLIS752 #752Diversity[QUOTE]@Sonaite @BakerChair @KaeliNWLib I think this pandemic has shown a spotlight on the digital divide that still exists. Schools are scrambling to provide ‘continuity of education’ when children don’t have access to devices and/or reliable Internet.”

Topic 7 (encouragement) accounted for 7.5% of the total tweets, where people express their support and inspire people to overcome difficulties together. One example of such a tweet is as follows:

“If you are working out in this scary world today, my love to you. If you are working from home, my love to you. If you are out of work, my love to you. If you are lonely, my love to you. If you are sick, my love to you. If you are grieving, my love to you. My love. To you.”

Topic 8 (back to office) contained the keywords “back” and “office” and accounted for 13.2% of the total tweets. Under this topic, people mostly tweet about their opinions toward the office, including those inquiring if the return to “office” will finally materialize or when people will be able to go back to office. Topic 9 (leasing) contains keywords “year,” “lease,” “renew,” and “office,” where people argue that some companies might not renew their office lease for the next year because of how well-suited WFH is for these companies.

**Figure 2:**
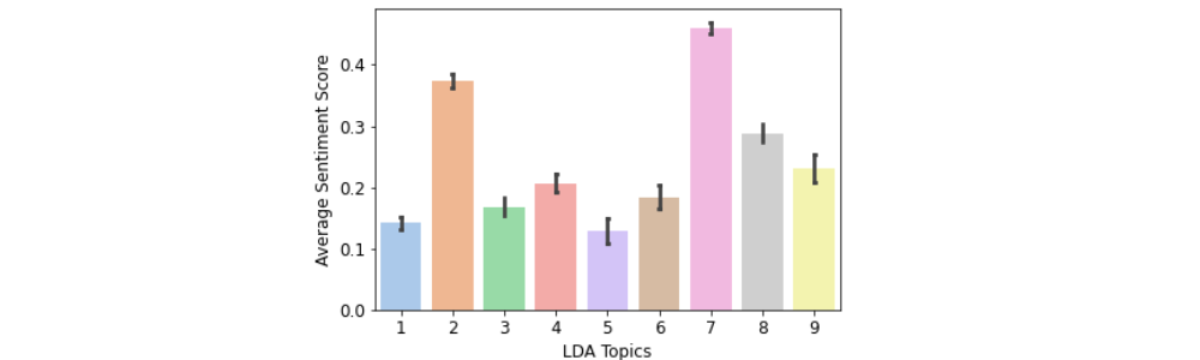
Average sentiment score of each topic. Topics are as follows: 1=family activities, 2=remote work or study, 3=quarantine, 4=dressing, 5=government and policy, 6=COVID-19 side effects, 7=encouragement, 8=back to office, and 9=leasing. LDA: latent Dirichlet allocation.

Figure 2 shows the average sentiment score of each topic. Topic 7 (encouragement) had the highest average sentiment score (0.460) and was considered the most positive topic; in contrast, topic 5 (government and policy) was the least positive topic (average sentiment score=0.129). As indicated above (“Feature Inference” subsection), the average sentiment score of all the users in our data set was 0.242. Among these 9 topics, all expressed a positive sentiment toward WFH. Moreover, topics 2 (remote work or study), 7 (encouragement), and 8 (back to office) had a sentiment score above the average, while topics 1 (family activities), 3 (quarantine), 4 (dressing), 5 (government and policy), 6 (COVID-19 side effects), and 9 (leasing) had a sentiment score below the average.

#### Money and Jobs are Discussed the Most When Government Accounts are Mentioned

In topic 5 (government and policy), “money” and “job” were the most prominent keywords churned by the LDA model. On further exploring the tweets under this topic, we found that a number of tweets mentioned government accounts and governor twitter accounts. An example of such a tweet is as follows:

“@SenBobCasey @SenToomey @GovernorTomWolf supply chain workers discouraged. Work from home pieces of the chain are essential. TEMPORARY layoffs making more than I am to wait to get called back to work, where’s the incentive to work? Why aren’t we included in stimulus 2.0?”

In addition, we also found some tweets about money, where people are somewhat worried about their financial status during the pandemic, such as losing money after being laid off from their jobs. Based on these findings, since April 2020 still marks the early stage of WFH, the economy would be a major priority for the government during this period.

#### Family Activities and Remote Work or Study Conflicts Among Age Groups

**Figure 3:**
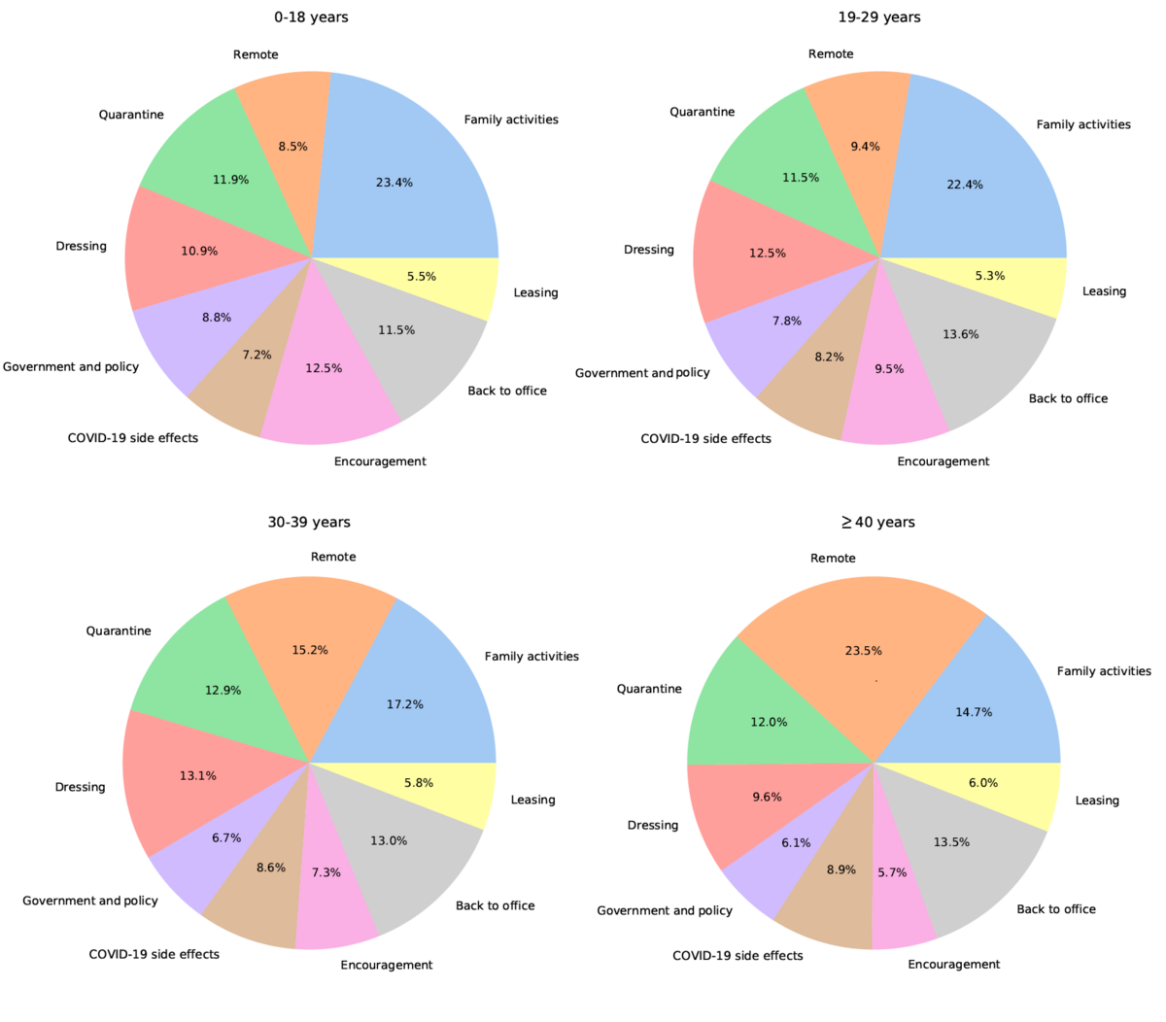
Topic distributions among different age groups.

As shown in Figure 3, as age increases, the proportion of people tweeting about remote work or study increased; moreover, lesser people tweeted about family activities. Among people aged 0-18 years, 23.4% of people tweeted about topic 1 (family activities) and 8.5% about topic 2 (remote work or study). In the age group of 19-29 years, 22.4% of people tweeted about family activities and 9.4% tweeted about remote work or study. Among people in their 30s, 17.2% tweeted about family activities and 15.2% tweeted about remote work or study. Among people older than 40 years, only 14.7% tweeted about family activities and 23.5% tweeted about remote work or study. Overall, topic 2 (remote work or study) is largely a work-related topic, which highlights work-family conflicts. Frone et al [35] reported that family boundaries are more permeable than work boundaries. Interestingly, based on our findings, we conclude that in the WFH environment, family boundaries are becoming more permeable among older people. On average, people aged ≥40 years accounted for 37.08% of the study population. However, under topic 1 (family activities), only 30.8% of the tweets were from people older than 40 years; however, under topic 2 (remote work or study), 52.1% of the tweets were from people aged ≥40 years. These interesting patterns are consistent with our findings that family-work boundaries are becoming weaker among older people.

#### Superwomen in WFH

**Figure 4:**
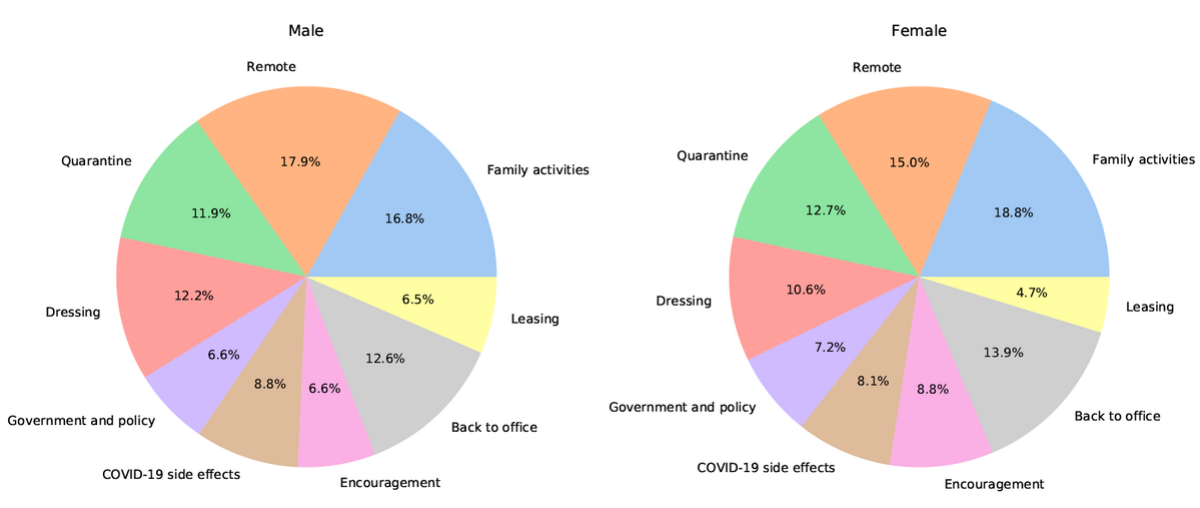
Topic distribution by gender.

As indicated above, women expressed more positive attitudes to WFH than men. Collins et al [31] reported that this might be because women tend to have more reduced work hours, and we confirmed this finding from the perspective of thematic analysis. In the goodness-of-fit test, we found that the topic distributions among men and women (Figure 4) were significantly different (*P*<.001). Based on the difference between topic distributions among men and women, we speculate that reduced work hours allow women to spend more time with their children and take care of their families. Topic 1 (family activities) is 1 of the topics to which women pay more attention. On an in-depth analysis of the tweets, we found that many tweets were about spending time with children while working from home. An example of such a tweet is as follows:

“That’d be me. I get to work from home and be with my kids. I’m loving every minute of this time with them!”

## Discussion

### Principal Findings

This study represents a large-scale quantitative analysis of public opinions on WFH in the United States during the COVID-19 pandemic. Through the lens of social media, we found that gender and age are the most influential features to public opinions about WFH. On performing ordinary least squares regression analysis, we found that the sentiment toward WFH varies across user characteristics. In particular, women are more positive about WFH, which could be related to the change in working styles [30] and reduced work hours compared to those of men [31]. People aged ≥40 years tend to be the most pro-WFH than other age groups. This could be owing to the fact that people of those ages are the most vulnerable to COVID-19, while also being the most difficult people to be re-employed upon losing their jobs. These people also need to work to mitigate the shrinkage of retirement savings that were invested in the rather inert stock market. People from high-income areas are more likely to have positive opinions about WFH, which echoes the findings of Barrero et al [3].

These nuanced differences are supported by a more fine-grained topic analysis. At a higher level, we found that all the topics expressed a positive sentiment about WFH. However, people expressed a more negative sentiment toward family activity and the government. Under the topic of family activity, we noticed that women pay more attention to family than men, and we identified superwomen in WFH. When people talk about government and policy, money and jobs are 2 major concerns. Furthermore, based on our analysis by age groups, we noticed that the family-work boundary is another issue that varies among different age groups. As age increases, more people prefer to discuss work rather than family, which implies that family boundaries are becoming more permeable than work boundaries.

### Implications

Barrero et al [3] reported that WFH would persist even after the pandemic. It is critical to understand public opinions on WFH to help improve their experience and to design a more suitable and flexible work policy. Our study suggests that there are nuanced differences across user characteristics. Government and company policymakers could design a more customized work policy to not only increase work productivity but also improve work satisfaction among their employees. It is also important to address the WFH-related disparities that have been reported among different racial and socioeconomic groups [36,37].

### Limitations

Our study is focused on the relationship between user characteristics and the sentiment about WFH. However, user occupation can be included in future analyses. Since the ability to WFH varies among different jobs [28], 1 potential hypothesis could be that people of different occupations have different opinions about WFH; thus, occupations would have an impact on the sentiment of WFH. In addition, there are some limitations of only using the Ethnicolr API to infer ethnicity. The Ethnicolr API is trained on voter registration data from Florida. First, using data from a single state (albeit a representative state) may not be ideal, since the pattern of names can be different among states. Another limitation is that our training data set was imbalanced (8,757,268 non-Hispanic White people, 1,853,690 non-Hispanic Black people, 2,179,106 Hispanic people, and 253,808 Asian people). Although these numbers are consistent with the population distribution in the United States, when training an inference model, we believe that the use of a more balanced data set could provide a better outcome.

### Conclusions

This is a large-scale social media–based study on people who are more likely to tweet about WFH. On performing ordinary least squares regression analysis, our study shows how the sentiment of WFH varies across user characteristics. On conducting a content-based analysis, we carried out an in-depth analysis to determine what Twitter users mainly discuss and how the content of their tweets correlates with the sentiment of WFH. This paper contributes to a better understanding of public opinions on WFH in the United States during the COVID-19 pandemic and contributes to making policies both at national and institution or company levels to improve the overall population’s experience of WFH.

## Abbreviations

API: application programming interface
LDA: latent Dirichlet allocation
VADER: Valence Aware Dictionary and Sentiment Reasoner
WFH: work from home
